# Human PBMC-based humanized mice exhibit myositis features and serve as a drug evaluation model

**DOI:** 10.1186/s41232-025-00365-6

**Published:** 2025-01-15

**Authors:** Akiko Nishidate, Kana Takemoto, Yuki Imura, Mikako Murase, Ryu Yamanaka, Manami Kikuchi, Junpei Anan, Sayuka Kato, Airi Akatsuka, Sachiko Mochizuki, Yuzo Koda

**Affiliations:** 1 Oncology & Immunology Unit, Research Division, Mitsubishi Tanabe Pharma Corporation, Kanagawa, 227-0033 Japan; 2 Discovery Technology Laboratories, Research Division, Mitsubishi Tanabe Pharma Corporation, Kanagawa, 227-0033 Japan

**Keywords:** Myositis, Polymyositis, Humanized mice, CD8^+^ T cells

## Abstract

Idiopathic inflammatory myopathies (IIMs) are a group of autoimmune disorders characterized by immune cell infiltration of muscle tissue accompanied by inflammation. Treatment of IIMs is challenging, with few effective therapeutic options due to the lack of appropriate models that successfully recapitulate the features of IIMs observed in humans. In the present study, we demonstrate that immunodeficient mice transplanted with human peripheral blood mononuclear cells (hPBMCs) exhibit the key pathologic features of myositis observed in humans and develop graft-versus-host disease. The hPBMC mice exhibit elevated serum levels of creatine kinase and aspartate transaminase, markers of myositis, and increased expression levels of myositis-related genes, such as vascular cell adhesion molecule 1, intercellular adhesion molecule 1, and serum amyloid A1, in muscle tissues. Histopathologic and flow cytometric analyses reveal the infiltration of CD8^+^ T cells in the muscle tissue of hPBMC mice, similar to that observed in patients with myositis, particularly in those with polymyositis. Transplantation of CD8^+^ T cell-depleted hPBMC leads to a significant reduction in polymyositis-like symptoms, in agreement with previous studies demonstrating CD8^+^ T cells as the main pathologic drivers of polymyositis. Furthermore, the transcriptome analysis of muscle tissue from hPBMC mice reveal extensive upregulation of characteristic genes of polymyositis, providing further support that hPBMC mice accurately reflect the pathophysiology of myositis in humans. Finally, administration of prednisolone or tacrolimus, which are commonly used for IIM treatment, leads to significant alleviation of myositis findings. Therefore, we propose that hPBMC mice should be considered as a model that accurately reflects the pathophysiology of myositis in human patients.

**Supplementary Information:**

The online version contains supplementary material available at 10.1186/s41232-025-00365-6.

## Background

Idiopathic inflammatory myopathies (IIMs) are a group of autoimmune disorders characterized by muscle inflammation, loss of muscle function, and pain, with unknown etiology. The estimated IIM incidence is 11–660 per 1,000,000 persons with a peak age of 50 years [1]. IIMs are classified into the following four main types based on clinicopathologic features: dermatomyositis, polymyositis, inclusion body myositis, and necrotizing autoimmune myositis. IIM cases positive for anti-aminoacyl tRNA synthetase antibodies, such as anti-Jo-1 antibodies, are considered to belong in a distinct subgroup known as anti-aminoacyl tRNA synthetase antibody syndrome [2]. In the USA, polymyositis and dermatomyositis are the most common IIM types [3]. Polymyositis is characterized by symmetric muscle weakness primarily affecting proximal muscles, whereas dermatomyositis presents with characteristic cutaneous symptoms in addition to muscle involvement. In addition to muscle and skin, IIMs can also involve other organs, such as joints, lungs, and heart, causing polyarthritis, interstitial lung disease, and myocardial damage, respectively, and may be associated with malignant tumors [4, 5]. Histopathologic IIM features in skeletal muscle include infiltration of immune cells, such as T and B cells and macrophages, around non-necrotic muscle fibers as well as muscle fiber degeneration and necrosis [6, 7]. Several studies reported the predominant presence of CD8^+^ T cells in the muscles of patients with polymyositis and the presence of CD4^+^ T cells and complement deposition in the muscles of patients with dermatomyositis, suggesting that muscle tissue damage by CD8^+^ T cells as a mechanism of polymyositis and autoantibody-mediated vascular damage in muscle as a mechanism of dermatomyositis [8–10]. However, subsequent studies revealed similarities in treatment response between polymyositis and dermatomyositis and the presence of dermatomyositis without myositis in some patients, suggesting that these two might be considered diseases presenting at different degrees within the same spectrum of inflammatory diseases causing myositis and dermatitis [11]. Immunosuppressive drugs, such as corticosteroids, tacrolimus, and intravenous immunoglobulin, are commonly used as therapeutic agents for IIMs [12]. Despite their ability to reduce inflammation, corticosteroids can promote muscle mass loss due to corticosteroid myopathy [13]. Importantly, approximately 20–45% of patients with IIM do not respond well to treatment and are at a high relapse risk [14], highlighting the need for more effective therapies.

The pathogenesis of myositis has long been associated with T-cell infiltration of muscle tissues, as supported by the clinical efficacy of tacrolimus, a potent inhibitor of T-cell activation, in patients with IIM [14, 15]. In attempts to investigate IIMs, several models of myositis have been developed, including experimental autoimmune myositis, which is induced by immunization with crude myosin, and C protein-induced myositis [16–20]. Other experimental models include the recapitulation of myositis and complicated lung symptoms by immunization with antigens targeted by myositis-specific autoantibodies, such as Jo-1, transcription intermediary factor 1γ, and melanoma differentiation-associated protein-5 [21–23], as well as the overexpression of MHC class I in muscle fibers to induce myositis [24]. However, these models do not fully recapitulate human disease, and the lack of models that accurately reflect pathophysiology and immune cell phenotypes observed in patients with IIM has hindered the development of effective therapeutic agents.

To address this gap, we aimed to elucidate the pathophysiologic changes that occurred in the muscles of mice with a humanized immune system, which was achieved with the transplantation of human peripheral blood mononuclear cells (hPBMC) or hematopoietic stem cells (HSC) in immunodeficient mice [25–28]. These mice would serve as a valuable tool in drug discovery efforts, improving our understanding of human immune response. In the present study, we focused on changes in the muscle tissue of mice with hPBMC transplantation to develop a model of myositis that closely reflected the pathology observed in humans. In this model, mice exhibited histopathologic findings of myositis, including elevated serum creatine kinase (CK) levels and human T-cell infiltration of muscle tissues. The transcriptome analysis of muscle tissue revealed genetic features resembling those of patients with polymyositis. Furthermore, tacrolimus and corticosteroids, commonly used in the clinical treatment of myositis, were effective in alleviating the muscle symptoms in this model. These findings suggest that hPBMC mice can serve as a novel model for studying myositis.

## Methods

### Mice

Female NSG mice (NOD.Cg-Prkdcscid Il2rgtm1Wjl/SzJ) were obtained at 5–6 weeks of age from the Jackson Laboratory Japan (Kanagawa, Japan) and used for hPBMC transplantation at 6–7 weeks of age. All mice were maintained under specific pathogen-free conditions in the Animal Care Facility of Mitsubishi Tanabe Pharma Corporation.

### Transplantation of human cells

Frozen human PBMCs were purchased from STEMCELL Technologies (Cancouver, BC, Canada). CD8^+^ T cell-depleted hPBMCs were obtained using magnetic beads coated with anti-human CD8 antibody with a MACS column (Miltenyi Biotec Japan, Tokyo, Japan), according to the manufacturer’s instructions. CD8^+^ T cell-depleted and complete hPBMCs were resuspended in phosphate-buffered saline and administered through the tail vein of NSG mice at a dose of 1 × 10^7^ cells/mouse to obtain hPBMCΔCD8T and hPBMC mice, respectively. As shown in Table 1, graft-versus-host disease (GVHD) symptoms were monitored using a clinical scoring system [29].

**Table 1:** Scoring criteria for GVHD

Grade	**0**	**1**	**2**
Weight loss	< 10%	10 to 25%	> 25%
Posture	Normal	Hunching noted only at rest	Severe hunching
Activity	Normal	Mild to moderately decreased	Stationary unless stimulated
Fur texture	Normal	Mild to moderate ruffling	Severe ruffling
Skin integrity	Normal	Scaling of paws/tail	Obvious areas of involved skin

### Isolation of mouse tissue-derived immune cells

Spleens and quadriceps muscles were collected from hPBMC and hPBMCΔCD8T mice. Spleens were mechanically dissociated with the gentleMACS dissociator (Miltenyi Biotec Japan) and lysed in HLB solution (Immuno-Biological Laboratories, Gunma, Japan) to remove red blood cells and obtain a suspension of mononuclear cells. Quadriceps muscles were enzymatically dissociated using the Skeletal Muscle Dissociation kit for mouse and rat (Miltenyi Biotec Japan), according to the manufacturer’s instructions.

### Flow cytometry

After blocking with an anti-FcR antibody (Human Trustain, BioLegend, San Diego, CA, USA) for 5 min, mouse tissue-derived immune cells were incubated with specific fluorescence-labeled antibodies at 4 °C for 20 min. The following antibodies and stains were used for flow cytometry: anti-human CD45 (PE/Cyanine7, clone 2D1; BioLegend), anti-human CD3 (BV605, clone UCHT1; BioLegend), anti-human CD8a (BV510, clone RPA-T8; BioLegend), anti-human CD4 (PerCP/Cyanine5.5, clone OKT4; BioLegend), and Fixable Viability Dye eFluor 780 (Thermo Fisher Scientific, Waltham, MA, USA). The following antibodies and stains were used for multiparameter flow cytometry: anti-human CD45 (BUV737, clone HI30; BD Biosciences, San Jose, CA, USA), anti-human CD3 (PE/Fire 640, clone SK7; BioLegend), anti-human CD8a (BV510, clone RPA-T8; BioLegend), anti-human CD4 (Spark Blue 550, clone SK3; BioLegend), anti-human leukocyte antigen (HLA)-DR (BUV395, clone L203.rMAb; BD Biosciences), anti-human CD16 (BUV496, clone 3G8; BD Biosciences), anti-human CD56 (BUV563, clone NCAM16.2; BD Biosciences), anti-human CXC motif chemokine receptor (CXCR) 3 (BV421, clone G025H7; BioLegend), anti-human CD200 (BV605, clone OX-104; BioLegend), anti-human CD127 (BV650, clone A019D5; BioLegend), anti-human CD45RA (BV711, clone HI100; BioLegend), anti-human ICOS (APC/Fire 750, clone C398.4A; BioLegend), anti-human CXCR6 (PE, clone K041E5; BioLegend), anti-human C–C chemokine receptor 2 (PE/Dazzle 594, clone K036C2; BioLegend), anti-human CD38 (PE/Fire 700, clone S17015A; BioLegend), anti-human programmed cell death protein 1 (PD-1: PE-Cy7, clone EH12.2H7; BioLegend), anti-human C–C chemokine receptor 7 (PE/Fire 810, clone G043H7; BioLegend), anti-human TIGIT (APC, clone A15153G; BioLegend), anti-human CXCR5 (Alexa Fluor 647, clone RF8B2; BD Biosciences), anti-human CD19 (APC/Fire 810, clone HIB19; BioLegend), anti-mouse CD45 (Spark NIR 685, clone 30-F11; BioLegend), and Zombie Dye NIR (BioLegend).

To detect intracellular cytokine production, tissue-derived cells were stimulated with 1 × Cell Stimulation Cocktail supplemented with protein transport inhibitors (Thermo Fisher Scientific) for 4 h. Next, the cells were permeabilized and fixed using the Foxp3/Transcription Factor Staining Buffer set (Thermo Fisher Scientific) and incubated with anti-human interferon (IFN)-γ antibody (APC, clone 4S.B3; BioLegend) for normal flow cytometry or with anti-human forkhead box protein P3 (FOXP3: PE-Cy5, clone 236A/E7; Thermo Fisher Scientific), anti-human Helios (FITC, clone 22F6; BioLegend), anti-human granzyme B (GZMB: PerCP-Cy5.5, clone QA16A02; BioLegend), antibodies for multiparameter flow cytometry. Normal flow cytometry was performed using a Fortessa X-20 (BD Biosciences) and analyzed using the FlowJo software (Tree Star, Ashland, OR, USA), whereas multiparameter flow cytometry was performed using an ID7000 Spectral Cell Analyzer (SONY Biotechnology, Tokyo, Japan) and analyzed using the Cytobank flow software (Beckman Coulter, Brea, CA, USA).

### Quantitative real-time polymerase chain reaction

Immediately after harvest, quadriceps muscles were stored in RNAlater (Thermo Fisher Scientific) at − 80 °C. RNA was isolated using TRIzol reagent (Thermo Fisher Scientific) according to the manufacturer’s instructions and immediately converted to complementary DNA (cDNA) using the High-Capacity RNA-to-cDNA kit (Thermo Fisher Scientific), according to the manufacturer’s instructions. For quantification, real-time polymerase chain reaction (PCR) was performed using the QuantStudio 7 Flex Real-Time PCR system (Thermo Fisher Scientific) with the TaqMan Universal Master Mix and the following predesigned probes (Thermo Fisher Scientific): vascular cell adhesion molecule 1 (*Vcam1*; Mm01320970_m1), intercellular adhesion molecule 1 (*Icam1*; Mm00516023_m1), and serum amyloid A1 (*Saa1*; Mm00656927_g1). Target gene expression levels were normalized to that of glyceraldehyde-3-phosphate dehydrogenase (*Gapdh*; Mm99999915_g1) in each sample.

### Measurement of serum enzyme levels

Serum levels of alanine aminotransferase (ALT) and aspartate aminotransferase (AST) were measured using a Fuji DRI-CHEM analyzer (FujiFilm, Tokyo, Japan), according to the manufacturer’s instructions. Serum Krebs on den Lungen-6 (KL-6) levels were measured using a mouse KL-6 enzyme immunosorbent assay kit (MyBioSource, San Diego, CA, USA), according to the manufacturer’s instructions. Serum CK levels were measured using the Fuji DRI-CHEM analyzer (FujiFilm) or the CK Activity Assay kit (Sigma-Aldrich, St. Louis, MO, USA), according to the manufacturer’s instructions. Plasma surfactant protein D (SP-D) levels were measured using a rat/mouse SP-D kit (Yamasa, Tokyo, Japan), according to the manufacturer’s instructions.

### Histology

Quadriceps muscles were fixed with 10% buffered formalin and embedded in paraffin. Sections prepared from the specimens were stained with hematoxylin and eosin or with anti-human CD8α antibody (M7103; Agilent Technologies, Santa Clara, CA, USA) or with anti-granzyme B antibody (EPR22645-206; Abcam, Cambridge, UK).

### RNA-Seq

RNA-Seq of muscle tissues was performed using RNA isolated for quantitative real-time PCR. The integrity and quantity of total RNA were evaluated using an Agilent 2100 Bioanalyzer RNA 6000 Nano kit (Agilent Technologies). Library preparation was performed using an NEBNext Ultra II Directional RNA Library prep kit (New England Biolabs, Ipswich, Massachusetts, USA) with the NEBNext Poly(A) mRNA Magnetic I NEBNext Poly(A) mRNA Magnetic Isolation module (New England Biolabs), according to the manufacturer’s instructions. The quality of the libraries was assessed using an Agilent 2200 TapeStation High Sensitivity D1000 assay (Agilent Technologies). The equally pooled sample libraries were sequenced using the Illumina NextSeq 500 (Illumina, San Diego, CA, USA) with 76-base-pair single-end reads. Sequencing adaptors, low-quality reads, and bases were trimmed with the Trimmomatic-0.39 tool [30]. Sequence reads were aligned to the mouse reference genome mm10 using STAR 2.7.9a [31]. The aligned reads were evaluated in downstream analyses using the StrandNGS 4.0 software (Agilent Technologies). Read counts allocated for each gene and transcript (Ensembl Database 2018.02.25) were quantified using the transcripts per million method [32, 33]. Principal component analysis (PCA plots, heatmap, and Pearson’s correlation matrices were generated using the R package. Gene Ontology enrichment analysis was performed using the clusterProfiler tool and R package.

### Statistical analysis

All statistical analyses were performed using SAS (version 9.04; SAS Institute, Cary, NC). Differences between the two groups were evaluated using a two-sided, unpaired Student’s *t*-test, and significance was accepted at a *p* value of < 0.05. Comparisons of more than two groups were performed using Williams’ multiple comparisons test and significance was accepted at a *p* value of < 0.025.

## Results

### hPBMC mice develop myositis in a CD8^+^ T cell-dependent manner

**Fig. 1:**
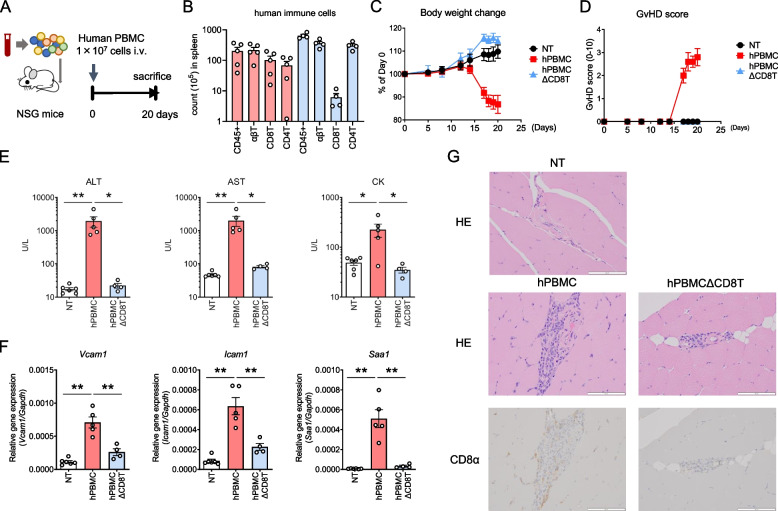
hPBMC mice develop myositis symptoms in a CD8^+^ T cell-dependent manner. NSG mice were intravenously administered hPBMC and sacrificed following the observation of GVHD-like symptoms or on day 20 after hPBMC administration. **A** Study design. Construction of hPBMC mice. **B** Number of human immune cells in spleen. Data are presented as means + standard error of the mean (SEM) (*n* = 4–6 per group). **C**–**E** Changes in body weight (**C**), GVHD score (**D**), and serum levels of ALT, AST, CK, and KL-6 (**E**). Data are presented as means ± SEM (*n* = 4–6 per group). *P < 0.05, **P < 0.01 by Student’s *t*-test. For statistical analyses of ALT, AST, and CK levels, logarithmic transformation was employed to equalize between-group variances. **F** Expression levels of *Vcam1*, *Icam1*, and *Saa1* in muscle. Data are presented as means ± SEM (*n* = 4–6 per group). *P < 0.05, **P < 0.01 by Student’s *t*-test. **G** Representative photomicrographs of muscle specimens stained with HE (upper panel) and IHC for CD8α (lower panel). NT, non-treated mice; hPBMC, human peripheral blood mononuclear cell; hPBMC mice, mice transplanted with hPBMC; hPBMCΔCD8T; mice transplanted with CD8^+^ T cell-depleted hPBMC; GVHD; graft-versus-host disease; ALT, alanine aminotransferase; AST, aspartate aminotransferase; CK; creatine kinase; KL-6; Krebs von den Lungen-6; HE; hematoxylin and eosin; and IHC; immunohistochemistry

Although previous studies reported that hPBMC mice exhibited T-cell-induced GVHD symptoms, the pathologic changes in muscle tissues have not been reported. Therefore, we evaluated the systemic and muscle-specific changes in immunodeficient mice transplanted with hPBMCs (hPBMC mice) (Fig. 1A). In addition, based on previous reports showing that CD8^+^ T cells caused the GVHD manifestations [34], we also evaluated the systemic and muscle-specific changes in immunodeficient mice transplanted with CD8^+^ T cell-depleted hPBMCs (hPBMCΔCD8T mice) to determine the contribution of the CD8^+^ T cells to the observed phenotype. We confirmed the engraftment of human PBMCs in both groups and the depletion of CD8^+^ T cells in pretransplant PBMCs and in hPBMCΔCD8T mice (Supplementary Fig. 1 and Fig. 1B).

Consistent with previous reports, hPBMC mice exhibited significant weight loss, worsening GVHD symptoms, and increased serum levels of ALT, a marker of liver injury, following PBMC transplantation compared to NT mice (Fig. 1D and E). Importantly, the serum levels of AST and CK, markers of muscle damage, were also increased in hPBMC mice compared to NT mice (Fig. 1E). The expression levels of genes indicating muscle damage, including *Vcam1*, *Icam1*, and *Saa1*, were significantly upregulated in muscle tissue compared to NT mice (Fig. 1F). Histopathologic analysis revealed human CD8^+^ T cell infiltration in the perifibrillar region of muscle tissues, resembling polymyositis (PM) in hPBMC mice (Fig. 1G).

Furthermore, not only systemic symptoms, such as body weight loss and GVHD, but also muscle symptoms, such as the serum levels of AST, CK, and genes indicating muscle damage were significantly more limited in hPBMCΔCD8T mice compared to hPBMC mice, indicating that the muscle symptoms developed in parallel with GVHD symptoms in a CD8^+^ T cell-dependent manner in hPBMC mice. In addition, we observed pulmonary symptoms, a major complication of myositis, in hPBMC mice. In the lungs, there was extensive infiltration of immune cells and an increase in plasma SP-D, a marker of lung damage (Supplementary Fig. 2). These results indicate that this model can detect pulmonary symptoms associated with myositis.

### Muscle tissue of hPBMC exhibit the characteristic gene signature of polymyositis in humans

**Fig. 2:**
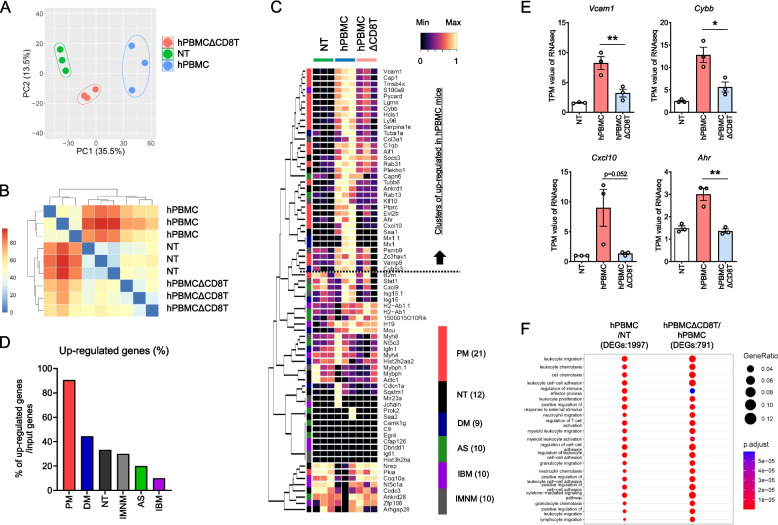
Muscle tissue of hPBMC mice exhibit the characteristic gene signature of polymyositis in humans. Gene expression profiles of the muscle tissue from NT, hPBMC, and hPBMCΔCD8T mice were evaluated using RNA-Seq (*n* = 3 per group). **A** Principal component analysis plot of top 2000 genes. **B** Pearson’s correlation matrix using top 75% of genes. **C** Heatmap of characteristic genes associated with various forms of myositis in specific tissues. **D** Percentage of characteristic genes of each myositis type in up regulated genes in hPBMC mice. **E** Expression of *Vcam1*, *Cybb*, *Cxcl10*, and *Ahr*, characteristic genes reported in patients with polymyositis, in muscle tissue of mice. Data are presented as means ± SEM. *P < 0.05, **P < 0.01 by Student’s *t*-test). **F** Gene ontology enrichment analysis showing the comparison of hPBMC mice with NT and hPBMCΔCD8T mice. DEG; differential gene expressions, PM; Polymyosits, NT; Normal muscle tissue, DM; Dermal myositis, AS; Antisynthetase syndrome, IBM; Inclusion body myositis, IMNM; Immune-mediated necrotizing myopathy

To investigate the global changes in gene expression in muscle tissues, we performed a comprehensive transcriptome analysis in hPBMC mice. The transcriptomes of hPBMC and hPBMCΔCD8T mice, and mice without hPBMC transplantation (NT mice) were compared using PCA and Pearson’s correlation matrix. The PCA analysis revealed that the transcriptomic changes observed in hPBMC mice compared to the NT mice were reversed in hPBMCΔCD8T mice, whereas Pearson’s correlation matrix revealed that the transcriptomes of the NT and hPBMCΔCD8T mice were comparable (Fig. 2A and B).

We also examined the expression patterns of genes associated with specific types of myositis reported in previous reports [35–37]. Cluster analysis revealed that the clusters upregulated in hPBMC mice included genes that were characteristic of both types of myositis (Fig. 2C and D). Of these, genes specific to PM were identified in clusters that were up regulated in hPBMC mice and accounted for more than 90% of the inputted genes (Fig. 2D). These results indicate that this model represents a system that reflects the pathogenesis of PM. Consistently, genes characteristic of patients with PM (e.g., *Vcam1*, *Cybb*, *Cxcl10*, and *Ahr*) were upregulated in hPBMC mice compared to NT mice and downregulated in hPBMCΔCD8T mice compared to hPBMC mice (Fig. 2C and E). By gene ontology enrichment analysis, the genes upregulated in hPBMC mice compared to NT mice were enriched in terms associated with immune system activation. Similar terms were observed in the comparison between hPBMCΔCD8T and hPBMC mice, suggesting that the enriched gene signatures in hPBMC mice were abolished by CD8^+^ T cell depletion (Fig. 2C and E). These results suggested that hPBMC mice accurately reflected the tissue gene signature of patients with IIMs and that this signature was associated with CD8^+^ T cell infiltration and activation.

### Muscle-infiltrating CD8^+^ T cells in hPBMC mice exhibit an activated phenotype

**Fig. 3:**
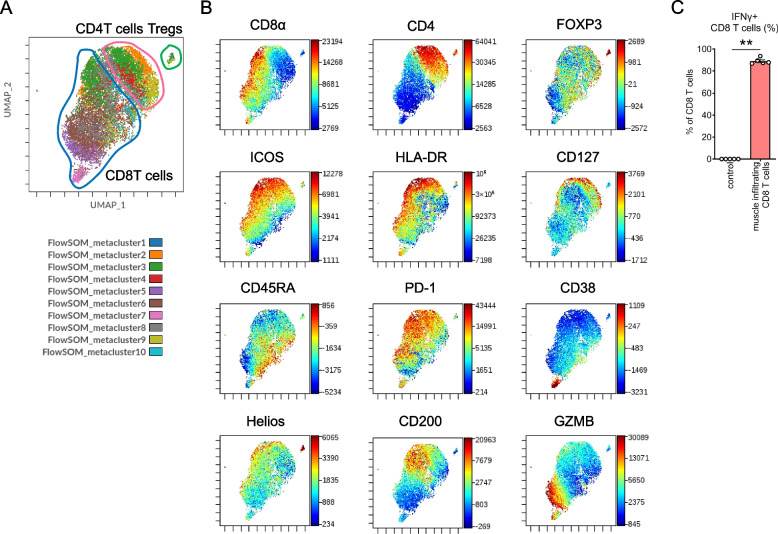
Muscle-infiltrating CD8^+^ T cells in hPBMC mice exhibit an activated phenotype. **A** Uniform manifold approximation and projection dimensional reduction and FlowSOM meta-cluster of muscle-infiltrating T cells. **B** Representative expression of surface and intracellular markers of T cells. **C** Intracellular staining for IFN-γ in the CD8^+^ T cells before PBMC transplantation (control) and in muscle-infiltrating CD8^+^ T cells. (*n* = 5 per group). Data are presented as means ± SEM. **P < 0.01 by Student’s *t*-test. IFN; interferon

Next, we determined the phenotype of CD8^+^ T cells in the muscle tissue of hPBMC mice using multiparameter flow cytometry. Based on 20-parameter flow cytometric analysis of CD3e^+^ T cells isolated from PBMCs in muscle tissue, dimensional reduction using uniform manifold approximation and projection revealed distinct clusters of CD8^+^ T cells (CD8a^+^/CD4^−^), CD4^+^ T cells (CD8a^−^/CD4^+^), and regulatory T cells (CD8a^−^/CD4^+^FOXP3^+^/Helios^+^) (Fig. 3A).

Among the CD8^+^ T cells, an activated CD8^+^ T cell cluster expressing PD-1, HLA-DR, and ICOS was identified within the CD45RA^−^ effector/memory population (Fig. 3B). Within the activated CD8^+^ T cell cluster, distinct subpopulations were observed based on the expression of CD38, GZMB, and CD200, suggesting the presence of distinct CD8^+^ T-cell subphenotypes. Similarly, among CD4^+^ T cells, an activated subpopulation expressing PD-1, HLA-DR, and ICOS was observed within the CD45RA^−^ effector/memory population, but cells expressing CD38, GZMB, or CD200 were not detected.

Furthermore, Intracellular cytokine staining revealed increased production of IFN-γ, a secretory activation marker, in CD8^+^ T cells isolated from the hPBMC in muscle tissues (Fig. 3C). In agreement with previous studies reporting that PD-1^+^/IFN-γ^+^/CD8^+^ T cells served as pathogenic cells in mouse models, our findings suggested that activated PD-1^+^/IFN-γ^+^/CD8^+^ T cells infiltrating muscle tissue induced damage in this model as well. Detailed analysis of multiparameter flow cytometry and intra-cellular staining of CD8^+^ T cells isolated from the spleen and peripheral blood are shown in Supplementary Figs. 3 and 4.

#### High correlation between muscle damage and muscle-infiltrating GZMB+CD8+ T cells in hPBMC mice

**Fig. 4:**
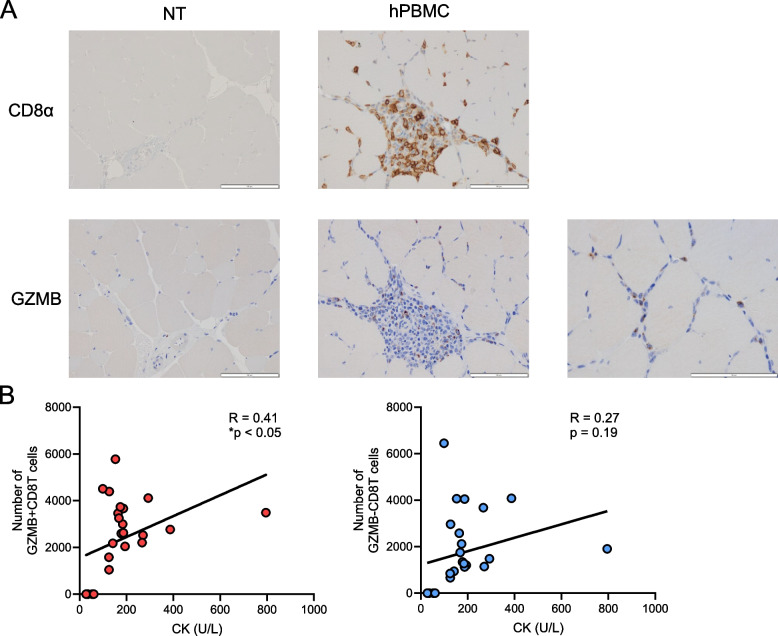
High correlation between muscle damage and muscle-infiltrating GZMB^+^CD8^+^ T cells in hPBMC mice. **A** Representative photomicrographs of muscle specimens stained with IHC for CD8α (left panel) and GZMB (right panel) of NT or hPBMC mice. **B** Scatter plot and correlation analysis between GZMB^+^CD8^+^ T cells or GZMB^−^CD8T^+^ cells in muscle tissue and CK of NT or hPBMC mice. *n* = 25 per group. *P < 0.05 by Pearson’s r test. GZMB; granzyme B

To examine the pathogenesis of myositis, we focused on the roles of different subsets of CD8 T cells using flow cytometry. Based on the analysis in Fig. 3, CD8 T cells infiltrating the muscles in this model were distinguished by the expression of GZMB, which prompted a histopathological examination of GZMB-expressing cells. We found that GZMB^+^CD8 T cells were distributed both in areas where lymphocytes accumulate and around the muscle fibers (Fig. 4A). The abundant infiltration of GZMB^+^CD8^+^ T cells in the perifibrillar region suggested their potential direct involvement in muscle cell injury. Next, we determined the correlation between GZMB^+^CD8^+^ T cells and GZMB^−^CD8^+^ T cells, which infiltrate muscle tissue, and the muscle damage marker CK. The results indicated a strong positive correlation between GZMB^+^CD8^+^ T cells and CK (Fig. 4B). Although a positive correlation was also observed between GZMB^−^CD8^+^ T cells and CK, it was not statistically significant. The results indicate that among CD8^+^ T cells in this model, GZMB^+^CD8^+^ T cells contribute more strongly to the observed pathogenesis.

### Symptoms of myositis in hPBMC mice improve with the administration of therapeutic drugs used for the treatment of IIM in humans

**Fig. 5:**
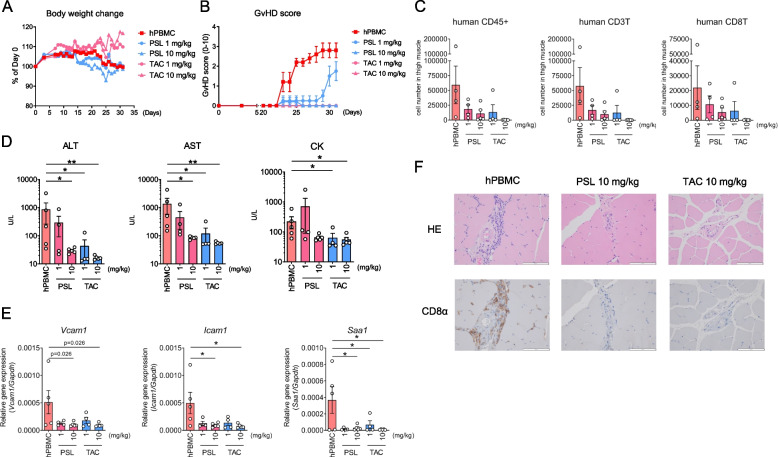
Symptoms of myositis in hPBMC mice improve with the administration of therapeutic drugs used for the treatment of IIM in humans. NSG mice were intravenously administered hPBMC and sacrificed following the observation of GVHD-like symptoms or on day 31 after hPBMC administration. Mice were orally administered PSL or TAC (1 or 10 mg/kg) from day 7 to day 31. **A** Changes in body weight from baseline. Data are presented as means (*n* = 4–5 per group). **B** GVHD score from baseline. Data are presented as means ± SEM (*n* = 4–5 per group). **C** Number of human immune cells in spleen. Data are presented as means ± SEM (*n* = 4–5 per group). **D** Serum levels of ALT, AST, CK, and KL-6. Data are presented as means ± SEM (*n* = 4–5 per group). *P < 0.025, **P < 0.005 by Williams’ multiple comparison test. For statistical analyses of ALT, AST, and CK levels, logarithmic transformation was employed to equalize between-group variances. **E** Expression of *Vcam1*, *Icam1*, and *Saa1* in muscle. Data are presented as means ± SEM (*n* = 4–5 per group). *P < 0.025 by Williams’ multiple comparison test. **F** Representative photomicrographs of muscle specimens stained with HE (upper panel) and IHC for CD8α (lower panel). IIM; idiopathic inflammatory myopathy, PSL; prednisolone, TAC; tacrolimus

Finally, we investigated whether hPBMC mice could be utilized for the preclinical evaluation of drug efficacy. Specifically, we examined the efficacy of oral administration of prednisolone and the calcineurin inhibitor tacrolimus, two commonly used therapeutics in patients with IIM, to determine whether drug intervention could improve disease symptoms and to validate hPBMC mice as a model of IIM. As shown in Fig. 5A, the administration of tacrolimus, but not prednisolone, improved body weight changes observed in hPBMC mice. However, both tacrolimus and prednisolone suppressed GVHD symptoms as assessed by clinical scoring (Fig. 5B). The numbers of CD8^+^ and CD4^+^ T cells infiltrating the muscle tissue were significantly reduced by the administration of tacrolimus but not prednisolone (Fig. 5C). Multiple markers of muscle damage, including serum AST and CK levels and *Icam1* and *Saa1* expression, exhibited significant improvements following treatment with tacrolimus and prednisolone (Figs. 5D and E). Additionally, histopathologic analyses revealed a marked improvement in muscle findings of inflammation with both tacrolimus and prednisolone administration (Fig. 5F).

Overall, these results demonstrated that the hPBMC mouse model reflected the pathophysiology of patients with IIM and could be used as a new model of drug discovery and screening.

## Discussion

The role of immunologic mechanisms in IIM pathogenesis has been extensively reported. Specifically, the pathologic analyses of human muscle tissue strongly suggest the involvement of T-cell infiltration in the affected tissue. In agreement, in the present study, our histopathologic analysis of muscle specimens from hPBMC mice indicated the involvement of T cells in these symptoms. In humans, the presentation of IIM includes the excessive activation of tissue-infiltrating CD8^+^ and CD4^+^ T cells and the increased expression of MHC class I in patients with polymyositis. The significant role of CD8^+^ T cell activation in the pathogenesis of myositis has been demonstrated previously [19, 22, 38]. Recent, detailed analyses of C protein-induced myositis models and pathologic findings in patients with IIM have revealed that PD-1^+^/CD8^+^ T cells induce tissue damage via interaction with PD-L1 and IFN-γ production in muscle tissue [39]. The hPBMC mouse model, albeit not an autologous immune response, exhibits human CD8^+^ T cell infiltration and activation in muscle tissue and recapitulates the importance of immune processes, including CD8^+^ T cell activation, in the pathogenesis of myositis. We also confirmed that the number of tissue-infiltrating PD-1^+^/CD8^+^ T cells was increased in hPBMC mice, consistent with previous reports [39]. Furthermore, these CD8^+^ T cells expressed other markers of activation, such as HLA-DR, and could be distinguished based on the expression of CD38, GZMB, and CD200. The finding that GZMB^+^CD8^+^ T cells contribute to the pathogenesis of this model is consistent with that of previous reports demonstrating the importance of GZMB^+^CD8^+^ T cells in the pathophysiology of human polymyositis [40]. Additionally, the clinical efficacy of approved drugs supports that the underlying pathology in this model is similar to that in human disease. In hPBMC mice, myositis is caused by human CD8^+^ T cells, highlighting the utility of this model as a more effective approach to evaluate the efficacy of drugs on human cells compared to conventional mouse models.

hPBMC mice exhibit characteristics of various types of myositis, but it has been shown to particularly reflect the pathophysiology of PM from a genetic and pathological perspective. In addition, the presence of PD-1^+^ and GZMB^+^ CD8^+^ T cells also indicates similarities with PM. In clinical settings, there is a co-occurrence of myositis GVHD (GVHD-myositis), and a contribution of PD-1^+^CD8^+^ T cells has been suggested [41]. Therefore, phenotypes shared between hPBMC mice, PM, and GVHD-myositis have been reported. Alternatively, the contribution of GZMB^+^CD8^+^ T cells to GVHD-myositis remains unclear, which suggests a potential difference in the role of hPBMC mice/PM in GVHD-myositis. In addition, the involvement of macrophages has been reported in GVHD-myositis. However, in hPBMC mice, the presence of transferred human macrophages is minimal (data not shown), and PM is primarily characterized by T cell-mediated pathophysiology, which suggests a difference in the contribution of macrophages between hPBMC mice/PM and GVHD-myositis. Pathologically, GVHD-myositis is characterized by fibrotic thickening of the fascia and fascial walls [42], which is not observed in the model/PM. Based on these observations, hPBMC mice exhibit similarities, particularly with PM, and represent a distinct pathology and mechanism from GVHD-myositis.

One limitation of the hPBMC mouse model is the infiltration of T cells to tissues beyond the muscle, causing GVHD symptoms. However, this issue may be addressed by genetic manipulation to allow for the expression of T cell-induced chemokines specifically in muscle tissue, thereby homing T cells to the muscle tissue. The use of humanized mice without GVHD symptoms, such as human HSC-transplanted immunodeficient mice, may allow for the proliferation of human B cells, which is not observed in hPBMC mice. However, a major challenge lies in approaches adopted to activate CD8^+^ T cells and induce myositis. Although B-cell engraftment and activation are observed, the human HSC model does not exhibit antigen–antibody responses [25–28]. Given that the T- and B-cell repertoires of donors are preserved in hPBMC mice, patient-derived PBMC transplantation might facilitate the activation of T cells and the deposition of human autoantibodies in the muscle tissue. Recent analyses of patient samples have revealed the significance of type I IFN signature, driving the development of therapeutic agents targeting this signaling pathway [12, 43]. Although we observed changes in IFN-related genes in the hPBMC mouse model, the modification of the type I IFN signaling, such as treatment with JAK inhibitors, did not have a significant effect on muscle symptoms (data not shown). Therefore, administration of agonists targeting toll-like receptors 3, 7, and 8 to upregulate IFN-α or the overexpression of IFN-α using adeno-associated virus-mediated methods may be considered to advance this model to more closely resemble the human disease, such as that utilized in mouse models of lupus nephritis [44–46].

## Conclusions

The hPBMC mouse model provides a platform that recapitulates mid/late phases of muscle pathology observed in patients with myositis, a condition with limited availability of appropriate models, illustrating its potential for drug discovery and screening.

## Supplementary Information

Additional file 1: Supplemental material

## Data Availability

The datasets analyzed during the current study are available in the supplemental materials.
